# A high-throughput screening RT-qPCR assay for quantifying surrogate markers of immunity from PBMCs

**DOI:** 10.3389/fimmu.2022.962220

**Published:** 2022-08-30

**Authors:** Daniel J. Browne, Ashton M. Kelly, Jamie L. Brady, Denise L. Doolan

**Affiliations:** Centre for Molecular Therapeutics, Australian Institute of Tropical Health and Medicine, James Cook University, Cairns, QLD, Australia

**Keywords:** HTS, PBMC, RT-qPCR, cost-effective, IFN-γ

## Abstract

Immunoassays that quantitate cytokines and other surrogate markers of immunity from peripheral blood mononuclear cells (PBMCs), such as flow cytometry or Enzyme-Linked Immunosorbent Spot (ELIspot), allow highly sensitive measurements of immune effector function. However, those assays consume relatively high numbers of cells and expensive reagents, precluding comprehensive analyses and high-throughput screening (HTS). To address this issue, we developed a sensitive and specific reverse transcription-quantitative PCR (RT-qPCR)-based HTS assay, specifically designed to quantify surrogate markers of immunity from very low numbers of PBMCs. We systematically evaluated the volumes and concentrations of critical reagents within the RT-qPCR protocol, miniaturizing the assay and ultimately reducing the cost by almost 90% compared to current standard practice. We assessed the suitability of this cost-optimized RT-qPCR protocol as an HTS tool and determined the assay exceeds HTS uniformity and signal variance testing standards. Furthermore, we demonstrate this technique can effectively delineate a hierarchy of responses from as little as 50,000 PBMCs stimulated with CD4^+^ or CD8^+^ T cell peptide epitopes. Finally, we establish that this HTS-optimized protocol has single-cell analytical sensitivity and a diagnostic sensitivity equivalent to detecting 1:10,000 responding cells (*i.e.*, 100 Spot Forming Cells/10^6^ PBMCs by ELIspot) with over 90% accuracy. We anticipate this assay will have widespread applicability in preclinical and clinical studies, especially when samples are limited, and cost is an important consideration.

## Graphical Abstract

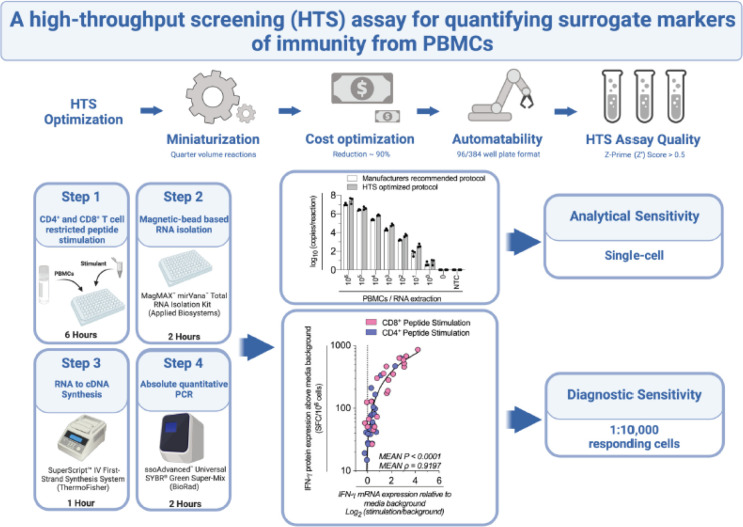

## Introduction

*Ex vivo* measurements of surrogate markers of immunity have informed immunobiological processes (1), provided disease biomarkers (2), and delivered measures of the effectiveness of candidate drugs and vaccines (3). These assays typically incubate peripheral blood mononuclear cells (PBMCs) in the presence of defined antigenic or mitogenic stimulants and quantitate protein production of effector molecules (*e.g.*, cytokines) using immunoassays such as flow cytometry or Enzyme-Linked Immunosorbent Spot (ELIspot) (4). ELIspot and flow cytometry both consume high-cost reagents (*e.g.*, monoclonal antibodies) and require relatively high numbers of PBMCs to achieve sufficient sensitivity (5, 6); especially when considering responses from sub-populations within PBMCs, such as antigen-reactive CD4^+^ or CD8^+^ T cells with relatively low precursor frequency (7). These factors pose severe constraints which preclude comprehensive immune evaluation and high-throughput screening (HTS) experiments.

PCR-based molecular diagnostics present a potential solution; in particular, reverse transcription quantitative-PCR (RT-qPCR), the gold-standard transcriptome-based assay (8), allows highly sensitive and specific *ex vivo* measurements of surrogate transcriptional markers of immunity from low numbers of PBMCs (9). However, due to costs and challenges associated with automation, RT-qPCR is generally considered a low throughput method (10). Ideally, an RT-qPCR-based HTS assay for quantifying surrogate markers of immunity would enable measurements from low numbers of PBMCs and use techniques that are cost-effective and amenable to both miniaturization and automation (11).

We have recently published a systematic evaluation of RNA extraction and reverse transcription kits to maximize the quantity and quality of isolated RNA and synthesized cDNA from human PBMCs (9). We found the mRNA expression of a key surrogate marker of immunity interferon-gamma (IFN-γ) correlated strongly to IFN-γ protein production measured by ELIspot (9). *Ex vivo* assays that quantify mRNA as a surrogate marker of immunity are typically limited by low genome-wide mRNA-protein correspondence rates (12). Nevertheless, certain classes of proteins, such as IFN-γ and other rapidly produced and secreted cytokines are much more highly correlated (9, 13), and therefore, may provide an mRNA target with comparable accuracy to protein-based immunoassays. Since each stage of this RT-qPCR assay is conducted in 96-well or 384-well format, the protocol is potentially suitable as an HTS assay. However, RT-qPCR is limited by cost, especially for studies involving many thousands of conditions that are typical of HTS (14).

To facilitate comprehensive HTS of surrogate markers of immunity from PBMCs, we present herein an HTS-optimized, highly sensitive and specific RT-qPCR protocol. This protocol reduces the cost of the RT-qPCR by almost 90%, is amenable to both miniaturization and automation, and achieved a ranking of excellent (Z’ factor >0.5 (15)) when evaluated for HTS uniformity and signal variance. When considering the analytical sensitivity (*i.e.* smallest number of cells detectable) and diagnostic sensitivity (*i.e.* smallest detectable response to stimulation) of our optimized protocol (16), we established single-cell analytical sensitivity and a diagnostic sensitivity equivalent to detecting 1:10,000 responding cells (*i.e.*, 100 SFC/10^6^ PBMCs by ELIspot) with over 90% accuracy. As a proof-of-concept for high-throughput *in vitro* PBMC functional testing, we applied this assay to investigate antigen-specific cytokine gene expression kinetics across 12 hours, with hourly resolution. Robust peptide-specific IFN-γ mRNA expression responses were detected between 3-9 hours post-stimulation, which we determined peaked at 6 hours post-stimulation when correlated to IFN-γ protein production across a larger number of peptides. This protocol provides a robust, scalable, and cost-effective RT-qPCR-based assay for high-throughput quantification of surrogate markers of immunity.

## Materials and equipment

### PBMC stimulatory reagents

-Phorbol 12-Myristate 13-Acetate (PMA), (Sigma-Aldrich)

-Ionomycin (Iono), (Sigma-Aldrich)

-Human Cytomegalovirus, Epstein Barr Virus, Polyomavirus, and Influenza virus Synthetic peptides (**Supplementary Table 1**).

### SYBR mastermix kits

-ssoAdvanced^™^ Universal SYBR^®^ Green Master-Mix (Bio-Rad)

### RNA extraction kits

-MagMAX^™^ *mirVana* ^™^ Total RNA Isolation Kit (Applied Biosystems)

### RNA to cDNA synthesis kits

-SuperScript^™^ IV First-Strand Synthesis System (ThermoFisher)

### Quantitative PCR primers

-PrimerBank^™^ primers (**Supplementary Table 2**)

### Antibodies

-anti-human IFN-γ monoclonal antibody (mAb) (Clone 1-D1K, MABTECH)

-anti-human IFN-γ biotinylated mAb (Clone 7-B6-1, MABTECH)

### Equipment

-QuantStudio 5 Real-Time PCR system (Applied Biosystems)

-AID ELIspot reader system (Autoimmun Diagnostika GmbH, Germany)

### Software

-QuantStudio Design and Analysis Software (v1.4.3, Applied Biosystems)

-ProcartaPlex Analyst Software (v1.0, ThermoFisher)

-GraphPad Prism (v7, GraphPad)

### Methods

### Samples

#### PBMCs

PBMCs from healthy donors were isolated by standard density gradient centrifugation and cryopreserved in 90%FBS/10%DMSO. Before use, samples were thawed rapidly at 37°C, treated with DNase I (100μg/mL; StemCell Technologies), and rested for 18 hours at 2x10^6^cells/mL in RPMI-1640 supplemented with 10% heat-inactivated AB human serum (Sigma-Aldrich), 100U/mL penicillin/streptomycin (ThermoFisher Scientific), 1xMEM non-essential amino acids (ThermoFisher Scientific), 2mM glutaMAX (ThermoFisher Scientific), 10mM HEPES (ThermoFisher Scientific), and 5x10^-5^M β-Mercaptoethanol (Sigma-Aldrich) (R10 media) at 37°C and 5% CO_2_. Viable PBMCs were counted with a CASY^™^ Cell Counter (OLS-OMNI Life Science).

#### Cell stimulation

Synthetic peptides (10ug/mL) representing well defined CD4+ and CD8+ T cell epitopes from Human Cytomegalovirus, Epstein Barr Virus, Polyomavirus, and Influenza virus (**Supplementary Table 1**) were tested alongside PMA/Iono (50ng/mL PMA, 1,000ng/mL Ionomycin) mitogen positive-control and a media-only negative-control. For RT-qPCR analysis; PBMCs were stimulated in 200μL R10 media in 96-well U-bottom plates. For IFN-γ ELIspot analysis, 4x10^5^ PBMCs per well were stimulated for 24 hours in 96-well ELIspot plates.

### RT-qPCR optimization

#### RNA extraction and reverse transcription

RNA was extracted as previously described (9), with MagMAX^™^ *mirVana* ^™^ Total RNA Isolation Kit (Applied Biosystems) following manufacturer’s instructions. Extracted RNA was converted to cDNA with SuperScript^™^IV First-Strand Synthesis System (ThermoFisher) following manufacturer’s instructions unless otherwise stated. For ‘Full Volume’ protocols, all reagents were used at the volume recommended by the manufacturer. For ‘Half Volume’ or ‘Quarter Volume’ protocol, all reagents were used at 50% or 25% of the volume recommended by the manufacturer, respectively. DEPC-Treated H_2_O (Invitrogen) was substituted to maintain equal reaction volume when evaluating presence, absence, or titration of reagents.

#### Quantitative PCR (qPCR)

qPCR was conducted as previously described (9). Briefly, mRNA copies/reaction were determined with absolute quantification based on a standard curve. *IFN-γ*, *TNF-α* and *IL-2* specific desalt-grade (Sigma-Aldrich) primers (**Supplementary Table 2**), obtained from PrimerBank^™^ (17) were used at 500nM using ssoAdvanced^™^ Universal SYBR^®^ Green Master-Mix (Bio-Rad). All reactions were run in technical triplicate in accordance with MIQE guidelines (18) at either 10μL or 5μL total volume. For 10uL reaction volumes, 1uL reverse transcription eluent diluted 1:2 in Ultra-Pure™ H2O (Invitrogen) was added per reaction. For 5uL reaction volumes, 1uL of reverse transcription eluent diluted 1:4 in Ultra-Pure™ H2O (Invitrogen) was added. Data was acquired using a QuantStudio5 Real-Time PCR system running QuantStudio Design and Analysis Software (v1.4.3, Applied Biosystems). Primer reaction efficiency was calculated by amplification of logarithmically diluted cDNA. A detailed final HTS-optimized RT-qPCR protocol is available **(** **Supplementary Protocol: Cost-Optimized Protocol** **).**

#### HTS uniformity and signal variance testing

Validation of uniformity and signal variance was conducted in accordance with the ‘HTS Assay Validation’ chapter of the National Institute of Health (NIH) ‘Assay Guidance Manual’ (19). Briefly, the coefficient of variation (CV) values and Z-Prime values were calculated from the mean and the standard deviation of the qPCR cycle threshold values for the ‘Min’, ‘Mid’ and ‘Max’ signals. *“Min” Signal* was the ‘media only’ stimulation, the *“Max” Signal* was ‘PMA/Iono stimulation’, and the “*Mid” Signals* was KGI and ARS peptide stimulations.

### Protein analysis

#### IFN-γ enzyme-linked immunospot (ELIspot) assay

IFN-γ ELIspot assays were performed as previously described (9). Briefly, 4x10^5^ PBMCs were plated in triplicate into 96-well MAIPS45-10 plates (Merck) and stimulated for 24 hours with or without peptide, PMA/Iono or media.

#### Analytical and diagnostic sensitivity testing

For determination of analytical sensitivity, mRNA was extracted from log_10_ serially diluted unstimulated PBMCs (10^6^-10^0^ cells/extraction) with a media-only extraction control processed in parallel. mRNA, cDNA synthesis and qPCR was conducted using either the manufacturers recommended protocol as previously described (9), or the HTS-optimized RT-qPCR protocol. Two strategies were tested for determining diagnostic sensitivity, where false negatives (FN) were considered mRNA values ≤0 or <1 where the matched ELIspot data was >0 or ≥100 respectively; and true positives (TP) were considered mRNA values >0 or ≥1 where the matched ELIspot data was >0 or ≥100 respectively. Assay accuracy was calculated as [(TP+TN)/(TP+TN+FP+FN)].

### Data analysis

The strength of the association between RT-qPCR *IFN-γ* mRNA gene expression and ELIspot IFN-γ protein expression was tested by Pearson’s correlation on log-transformed data. *P* values and Pearson’s correlation coefficient (ρ) were reported. Analytical sensitivity was analyzed with log_10_ transformed data using a repeated-measures two-way ANOVA. GraphPad Prism version 8.3.0 (GraphPad Software) was used and *P* values <0.05 were considered statistically significant.

## Results

### A high correlation between IFN-γ mRNA and IFN-γ protein ELIspot quantification persisted following RT-qPCR reagent miniaturization

To develop a sensitive and specific HTS tool to quantify surrogate markers of immunity from PBMCs, we first stimulated either 4x10^5^, 1x10^5^ or 0.5x10^5^ PBMCs with CD4^+^ T cell peptide epitopes. *IFN-*γ mRNA expression was quantified by RT-qPCR (9) and correlated with IFN-γ protein production quantified by ‘gold-standard’ ELIspot. When tested with a Spearman’s rank correlation coefficient (ρ), we found a high correlation between mRNA and protein, which decreased with stimulated cell number (**Supplementary Figure 1A**). When correlating *IFN-*γ mRNA expression with IFN-γ protein production, we modelled logarithmically (Log_2_) transformed mRNA data against linear protein data presented on a logarithmic scale (20). Log_2_ transformation allowed visualization of protein measurements approaching the limit of ELIspot sensitivity (*i.e.*, <100 SFC/10^6^ PBMCs) (21). This graphical presentation did not change the ρ or *P* values (**Supplementary Figure 1B**). Given the high correlation between gene and protein expression observed, we chose to progress with testing using 1x10^5^ stimulated PBMCs.

**Figure 1:**
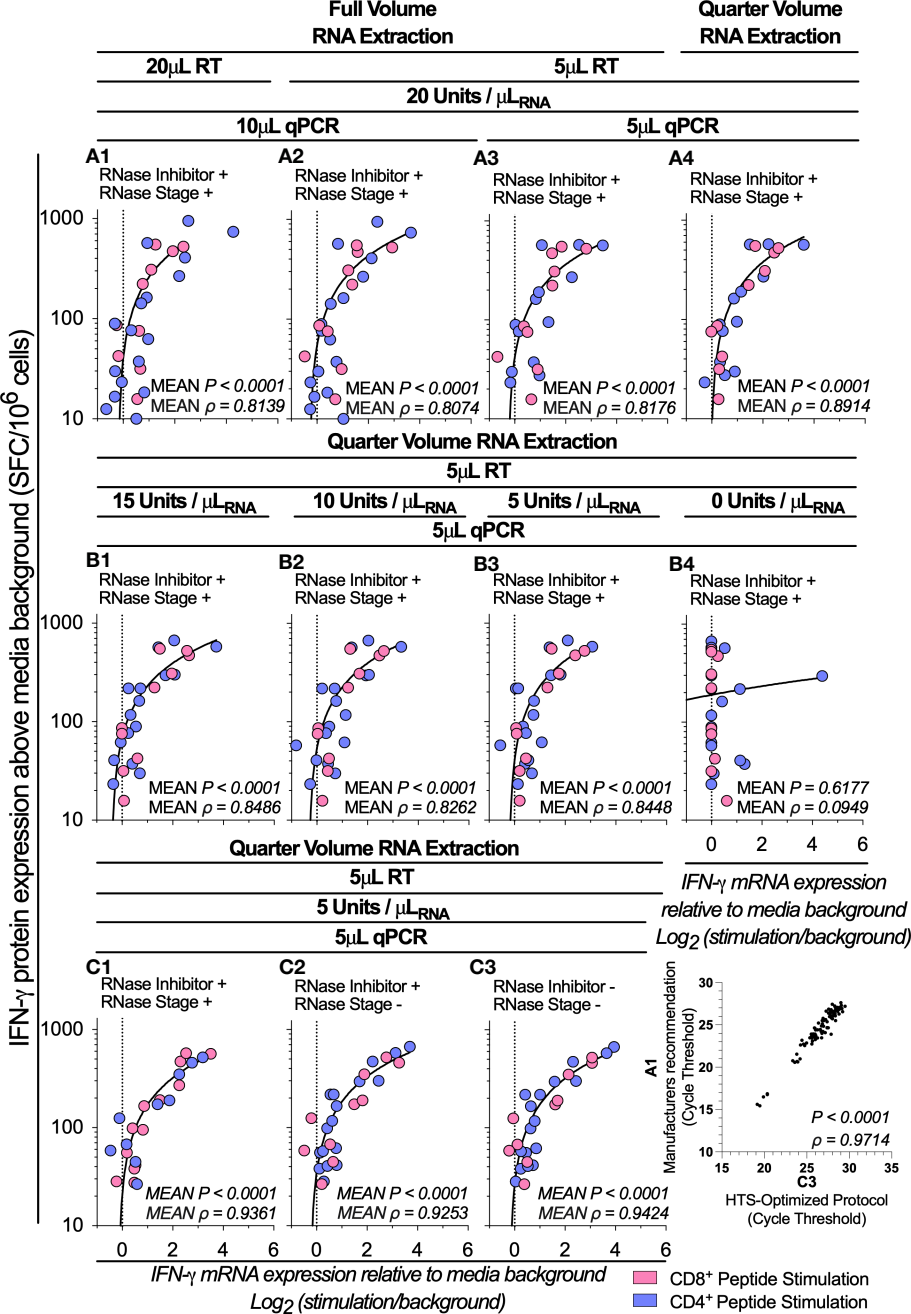
Assay optimization. IFN-γ mRNA expression by RT-qPCR correlated to IFN-γ protein production by ELIspot across various test conditions denoted **A1-A4, B1-B4** and **C1-C3** following stimulation of PBMCs (1x10^5^; n=5) with peptides representing two well-defined CD4^+^ (Blue Dots) T cell peptide-epitopes (Influenza_57-71_ KGILGFVFTLTVPSE and Influenza_260-284_ ARSALILRGSVAHKSCLPACVYGP) and two CD8^+^ (Pink Dots) T cell peptide-epitopes (Influenza_58-76_ GILGFVFTL and Epstein Barr Virus_280-288_ GLCTLVAML). Conditions **A1-A3** were evaluating the correlation between RNA isolation, cDNA synthesis and qPCR reagent miniaturizations; **B1-B4** were evaluating the correlation between reverse transcription (RT) reactions containing the Superscript IV enzyme at a concentration of 15, 10, 5, and 0 units/μL_RNA_; and **C1-C4** were evaluating the correlation between reverse transcription (RT) reactions including RNase Inhibitors and a post-cDNA synthesis RNase digestion stage, and reactions excluding the RNase digestion stage; and RT reactions excluding the RNase digestion stage and RT reactions excluding both the RNase digestion stage and RNase Inhibitors. Shown are the mean gene copy number of technical triplicate RT-qPCR assays correlated to mean of triplicate IFN-γ spot forming cells (SFC) by ELIspot, with both mRNA and protein measurements corrected for background. The technical means of qPCR cycle threshold values (Ct value) combined (for all tests and controls) were correlated between test conditions **A1** and **C3**. ELIspot data <10 SFC/10^6^ were graphically omitted. The strength of each association was tested by Pearson’s correlation on log-transformed data, with* P* values and Pearson’s correlation coefficient (ρ) reported.

We next sought to systematically reduced the volume and concentration of the reverse transcription (RT), qPCR and RNA isolation reagents. We therefore stimulated 1x10^5^ PBMCs with a portfolio of well-characterized CD8^+^ and CD4^+^ T cell restricted peptide epitopes and evaluated IFN-γ mRNA expression from quarter volume RT reactions and RNA isolations, and from 5μL total volume qPCR reactions. When considering the mean ρ of the triplicate replicates, mRNA expression retained a high correlation with protein expression at all conditions tested (**Figure 1** (A1): *P*<0.0001, ρ=0.8139; vs **Figure 1** (A4): *P*<0.0001, ρ=0.8914). When considering the ρ of the individual triplicate experimental technical replicates, multiple comparisons testing found a significantly increased correlation between IFN-γ mRNA and protein following the use of quarter-volume RNA extractions (A1 vs A4 *P=0.0110*, **Supplementary Figure 2A**). When considering the cycle threshold value of all measured samples (*i.e.*, inclusive of controls), the RT-qPCR protocol measurements correlated highly between all test conditions (ρ>0.98, **Supplementary Figure 2A**). Overall, these data demonstrate that all stages of our RT-qPCR protocol are amenable to miniaturization without loss of sensitivity.

### A high degree of correlation between mRNA and protein was maintained following a four-fold reduction in the concentration of SuperScript^™^IV RT enzyme

We next sought to determine whether reducing the concentration of the SuperScript^™^IV RT enzyme impacted the correlation between *IFN-γ* mRNA and IFN-γ protein quantification. We titrated the SuperScript^™^IV RT enzyme concentration from 15 to 5 units/μL_RNA_. We found the correlation between the RT-qPCR protocol and ELIspot was statistically significant across all RT reactions which contained enzyme (**Figure 1** (B3): *P*<0.0001, ρ=0.8448). When considering the ρ of the triplicate experimental technical replicates, reducing the concentration of the RT enzyme did not impact the high correlation between IFN-γ mRNA and protein (**Supplementary Figure 2B**). When considering the cycle threshold value of all measured samples, the RT-qPCR protocol measurements correlated highly between all tested conditions which contained enzyme (10 vs. 5 units/μL_RNA_: *P*<0.0001, ρ=0.9969; **Supplementary Figure 2B**), but not against the no-enzyme control (5 vs. 0 units/μL_RNA_: *P=*0.1072, ρ=0.1681; **Supplementary Figure 2B**). These data demonstrate that highly accurate measurements of epitope-specific *IFN-γ* mRNA stimulatory responses can be achieved with RT reactions containing the SuperScript^™^IV enzyme at a concentration as little as 5 units/μL_RNA_.

### RNase inhibitors and RNase treatment are not required to maintain a high correlation between mRNA and protein measurements

We next sought to investigate the impact of RNase inhibitors and post-cDNA synthesis Ribonuclease H (RNaseH) digestion on the correlation between IFN-γ mRNA and IFN-γ protein measurements. When stimulating 1x10^5^ PBMCs with a range of CD8^+^ and CD4^+^ T cell restricted peptide epitopes, we found the correlation between the RT-qPCR protocol and ELIspot was statistically significant across all conditions regardless of the presence of RNase inhibitor or RNaseH [*P*<0.0001, ρ=0.9424; **Figure 1** (C3)]. When considering the ρ of the triplicate experimental technical replicates, there was no statistically significant change in the correlation between IFN-γ mRNA and protein either when the RNase digestion was eliminated or when the RNase inhibitors were absent (**Supplementary Figure 2C**); and the cycle threshold values of all measured samples correlated highly between all tested conditions (ρ>0.99; **Supplementary Figure 2C**). These data demonstrate that omitting RNase inhibitors or RNaseH had no impact on the correlation between IFN-γ mRNA and protein measurements. When considering the cycle threshold value of all measured samples (*i.e.*, inclusive of controls), the RT-qPCR protocol measurements correlated highly between the manufacturers recommended protocol and the HTS-optimized protocol (*P*<0.0001, ρ=0.9714; **Figure 1** A1 vs C3). Taken together, the protocol miniaturization and modifications described above resulted in a reduction of the overall cost of the RT-qPCR by almost 90%.

### HTS assay quality assessment

We next sought to assess the uniformity and signal variability of this optimized assay to demonstrate its suitability as an HTS tool. We stimulated 1x10^5^ PBMCs with CD4^+^ T cell peptide epitopes in technical triplicate inter-day tests and compared IFN-γ mRNA expression as measured with the above-optimized protocol to IFN-γ protein production measured by ELIspot. The correlation between the RT-qPCR protocol and ELIspot was statistically significant across all three technical replicates (**Supplementary Figure 3**). When validating the uniformity and signal variance between these replicates, all tested coefficient of variation (CV), values were well below the NIH’s 20% acceptance criteria threshold (between 1.20%-1.49%, 2.48%-4.45% and 1.02%-2.60% for the *Min*, *Mid* and *Max* signals, respectively; **Table 1**). The Z-prime score (Z’) of the replicates ranged from 0.548-0.630, all above the NIH’s 0.5 threshold for ‘Excellent HTS Assay’ (15). These data demonstrate that this assay has uniformity and signal variability that passes the initial HTS assay quality assessment.

**Table 1:** Assay uniformity and quality assessment.

	MEDIA ONLY (*Min Signal*)			KGI (*Mid Signal 1*)			ARS (*Mid Signal 2*)			PMA/Iono (Max *Signal*)			
EXPT	Mean	SD	CV_(%)_	Mean	SD	CV_(%)_	Mean	SD	CV_(%)_	Mean	SD	CV_(%)_	Z’
1	28.239	0.671	1.37%	26.697	2.056	4.45%	26.959	1.626	3.48%	19.937	0.353	1.02%	0.630
2	28.454	0.732	1.49%	26.445	1.716	3.75%	27.365	1.173	2.48%	18.364	0.827	2.60%	0.548
3	27.088	0.563	1.20%	25.318	1.148	2.62%	26.253	1.542	3.39%	17.466	0.564	1.86%	0.627

Mean: Mean of cycle threshold value (Ct); SD: Standard deviation of Ct values; n=5.

Pass: Coefficient of variation (CV) < 20%; Z-prime score (Z’) > 0.4.

### The highest magnitude of response following peptide epitope stimulation occurs between 3- 6-hours post-stimulation

**Figure 2:**
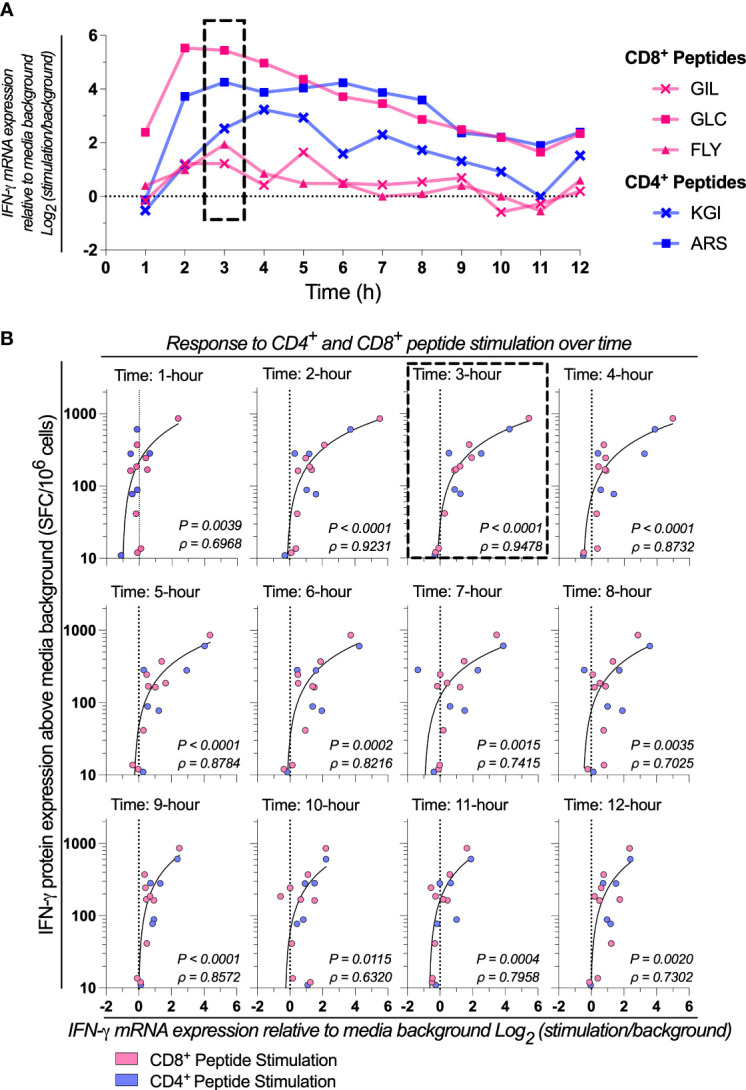
Response to CD4^+^ and CD8^+^ peptide stimulation over time. **(A)** IFN-γ mRNA expression by absolute-quantitative HTS-optimized RT-qPCR in response to stimulation with two CD4^+^ (Influenza_57-71_ KGI and Influenza_260-284_ ARS) and three CD8^+^ (Influenza_58-76_ GIL, Epstein Barr Virus_356-364_ FLY and Epstein Barr Virus_280-288_ GLC) peptides representing well-defined T cell epitopes across 1-12 hours post-stimulation. Shown is a representative sample. **(B)** IFN-γ mRNA expression by RT-qPCR correlated to IFN-γ protein production by ELIspot in response mRNA expression. Single RNA extractions, with single reverse transcription reactions per n (n=3) per stimulation were performed, with qPCR and ELIspot performed in technical triplicate. Technical mean of gene copy number or spot forming cells (SFC) corrected for background are shown. The strength of the association between RT-qPCR *IFN-γ* mRNA gene expression and ELIspot IFN-γ protein expression was tested by Pearson’s correlation on log transformed data. ELIspot data <10 SFC/10^6^ were graphically omitted. The 3-hour timepoints are highlighted (dashed box). P values and Pearson’s correlation coefficient (ρ) reported.

We next sought to determine the optimal time point post-stimulation for correlating mRNA expression to protein production by assessing the kinetics of cytokine expression. We evaluated the kinetics of IFN-γ, *IL-2*, and *TNF-α* mRNA expression each hour across 12 hours from 1x10^5^ PBMCs stimulated with a range of CD8^+^ and CD4^+^ T cell restricted peptide epitopes. Donor-specific peak IFN-γ mRNA expression was peptide-specific (**Figure 2A**). The profile of *IL-2* mRNA expression was similar to IFN-γ, but the *TNF-α* peak tended to be slightly delayed (**Supplementary Figure 4**). When correlating *IFN-*γ mRNA expression to IFN-γ protein production by ELIspot, we found a high correlation was retained across many time points, with the highest occurring at the 3 hours post-stimulation timepoint (3-hours: *P*<0.0001, ρ=0.9478; **Figure 2B**). These data suggested that although no single timepoint is optimal for all peptides, as peak IFN-γ mRNA expression is donor and peptide-specific, the highest magnitude of IFN-γ mRNA response to peptide stimulation (*i.e.*, expression relative to media background) generally occurs between 3- and 9-hours post-stimulation.

### The HTS-optimized protocol has single-cell analytical sensitivity and a diagnostic sensitivity equivalent to at least 100 SFC/10^6^ by ELIspot

**Figure 3:**
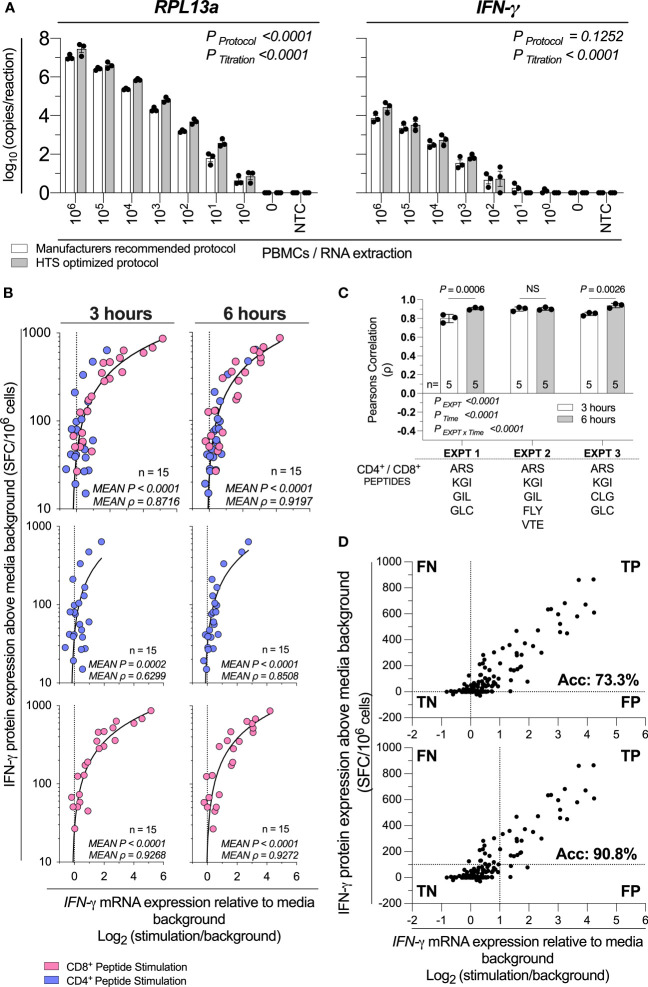
HTS-optimized RT-qPCR analytical and diagnostic sensitivity. **(A)** Assay analytical sensitivity of IFN-γ and RPL13a mRNA copies per reaction from log_10_ dilutions of unstimulated PBMCs from 10^6^ to 0. Samples were tested alongside blank extraction control (0) and qPCR no template control (NTC). mRNA expression was determined by absolute-quantitative RT-qPCR with manufacturer’s recommended protocol (White Bars) or HTS optimized protocol (Grey Bars), with gene copy number per reaction normalized to log_10_ copies per reaction. Biological replicates (n = 3), single RNA extractions, with single reverse transcription reactions per extraction were performed. Sample mean calculated from the mean of the technical triplicate qPCR reactions. Biological mean ± biological SEM shown. Significant differences due to protocol or PBMC titration were analyzed by two-way ANOVA. **(B)** IFN-γ mRNA expression by RT-qPCR correlated to IFN-γ protein production by ELIspot across 3-hours or 6-hours post-stimulation of PBMCs (1x10^5^; n=15) stimulated with peptides representing two well-defined CD4^+^ T cell peptide-epitopes (Influenza_57-71_ KGI and Influenza_260-284_ ARS; blue) and CD8^+^ T cell peptide-epitopes (Influenza_58-76_ GIL and Epstein Barr Virus_280-288_ GLC, Epstein Barr Virus_300-309_ FLY, Epstein Barr Virus_300-309_ VTE, or Epstein Barr Virus_300-309_ CLG; pink). Shown are the mean gene copy number of technical triplicate RT-qPCR assays correlated to the mean of triplicate IFN-γ spot forming cells (SFC) by ELIspot; then data separated by CD4^+^ or CD8^+^ restriction. Data from three independent inter-day experiments, with both mRNA and protein measurements corrected for background. ELIspot data <10 SFC/10^6^ were graphically omitted. The strength of each association was tested by Pearson’s correlation on log-transformed data, with* P* values and Pearson’s correlation coefficient (ρ) reported. **(C)** The RT-qPCR assay was performed in technical triplicate, with each replicate individually correlated to the mean IFN-γ SFC by ELIspot, with the ρ shown. The technical variability of ρ between inter-day experiments (EXPT), the 3- or 6-hour timepoints (Time), and their interaction (EXPT x Time) was tested with a Two-Way ANOVA with a Bonferroni corrected multiple comparisons test. Shown are the technical mean ± technical SEM; and the peptides used in each experiment. P > 0.05 were considered non-significant (NS). **(D)** A confusion matrix demonstrating true-positive (TP), false-positive (FP), true-negative (TN) and false-negative (FN) rate of the HTS-optimized RTqPCR protocol relative to ELIspot results. Data are inclusive of all above CD4^+^ and CD8^+^ peptide stimulation responses and additional (n=3) samples stimulated with (30x) CD8^+^ T cell peptide-epitopes. Shown are the mean gene copy number of technical triplicate RT-qPCR assays correlated to the mean of triplicate IFN-γ spot forming cells (SFC) by ELIspot.

Finally, we sought to investigate the analytical sensitivity (*i.e.* smallest number of cells detectable) and diagnostic sensitivity (*i.e.*, smallest detectable response to stimulation (16)) of this HTS-optimized assay. To assess the analytical sensitivity, RNA was extracted from a log_10_ serial dilution of unstimulated PBMCs, and the expression of IFN-γ and the reference gene *60S ribosomal protein L13a* (RPL13a) were determined using the HTS-optimized protocol in comparison to the manufacturer’s recommended protocol (**Figure 3A**). *RPL13a* expression was detected in all tested biological replicates at the single-cell level in both protocols. These data establish that the HTS-optimized protocol can quantify RNA to the single-cell level. IFN-γ expression was detected when extracted from cell numbers typical of PBMC stimulation assays for both protocols (10^6^-10^4^ PBMCs per stimulation; **Figure 3A**) (9).

To assess the diagnostic sensitivity of the assay, we stimulated 1x10^5^ PBMCs with a range of CD8^+^ and CD4^+^ T cell restricted peptide epitopes, and compared IFN-γ mRNA expression from 3-hours and 6-hours to IFN-γ protein production measured by ELIspot. Data were pooled from three inter-day experiments, each performed in technical triplicate replicate. The combined mRNA/protein correlation was statistically significant (*P<*0.0001, **Figure 3B**) at 3-hour and 6-hour time points in agreement with previously acquired data. The combined CD4^+^ and CD8^+^ peptide mRNA/protein correlation were highest at the 6-hour time point (ρ=0.8716 vs ρ=0.9197; 3 hours vs 6 hours; **Figure 3B**). Additionally, we found the RTqPCR true-positive rate (*i.e.*, inverted ratio of false-negative (FN) RTqPCR results relative to the true-positive (TP) RTqPCR results) compared to ELIspot at 3- and 6-hours crossed 95% (*i.e.*, FN<5% of total RT-qPCR results) at 50.8 and 41.7 SFC/10^6^ PBMCs respectively (**Supplementary Figure 5**). When considering the ρ of the inter-day technical replicates, the IFN-γ mRNA and protein correlation varied between experiments and time (*P*_EXPT_<0.0001; *P*_Time_<0.0001; **Figure 3C**). The interaction was also statistically significant (*P_EXPTxTime_* <0.0001; **Figure 3C**), which demonstrates the optimal timepoint for correlating IFN-γ mRNA expression and protein production is both donor- and peptide-specific. However, when considering large numbers of peptides and samples, these data demonstrate that 6-hours post-stimulation produces a marginally higher correlation.

To determine the diagnostic sensitivity of the HTS-optimized RT-qPCR protocol, we stimulated 1x10^5^ PBMCs with 30 CD8^+^ cell restricted peptide epitopes and compared IFN-γ mRNA expression from 6-hours to IFN-γ protein production measured by ELIspot (**Supplementary Figure 6**) and combined these data with all above qPCR and ELIspot responses when RTqPCR data were collected with the HTS-optimized protocol. When considering a threshold of positivity as responses greater than 0, the assay accuracy was calculated as 73.3% (**Figure 3D**). When considering IFN-γ protein expression equivalent to or greater than 100 SFC/10^6^ PBMC, and a log_2_ relative increase of IFN-γ mRNA equivalent to or greater than 1 (*i.e.*, a doubling of IFN-γ mRNA expression), the assay accuracy was calculated as 90.8% (**Figure 3D**). Taken together, these data establish that our HTS-optimized protocol has single-cell analytical sensitivity and a diagnostic sensitivity conservatively estimated to be at least equivalent to detecting 1:10,000 responding cells (*i.e.*, 100 SFC/10^6^ PBMCs) with 90% accuracy.

## Discussion

This report describes an HTS-compatible RT-qPCR-based assay specifically designed to provide a high-throughput, robust, scalable, and cost-effective alternative to protein-based *in vitro* immunoassays. With this protocol, the cost per sample has been reduced by almost 90% compared to standard practice, and the assay consumes 10-300 fold fewer PBMCs than commonly used immunoassays (9). This assay has single-cell analytical sensitivity and a diagnostic sensitivity capable of detecting 1:10,000 responding cells with an accuracy greater than 90%. We demonstrate a very high mRNA/protein correlation between our HTS-optimized RT-qPCR protocol and ELIspot. ELIspot is often considered the ‘gold-standard’ PBMC immunoassay (22) and has been extensively optimized for global consistency as part of the HIV/AIDS Comprehensive Cellular Vaccine Immune Monitoring Consortium (CCVIMC) (23). Our RT-qPCR-based protocol effectively delineated a hierarchy of IFN-γ stimulation responses for different CD4^+^ and CD8^+^ T cell peptide epitopes and defined 6-hours post *in vitro* stimulation as the optimal timepoint for IFN-γ immune readout. However, this may not be universal for all cytokines, and other target surrogate markers of immunity should be validated independently.

Our assay was optimized for analysis using cryopreserved PBMCs since these are common sample sources for human immunoassays (24), as blood collection is less-invasive (25) and PBMC isolation is relatively technically straightforward (26) and cost-efficient (27). Additionally, cryopreserved PBMCs can be shipped globally (28) for batched testing (29) or long-term storage in biobanks (30). It is reasonable to expect an even higher diagnostic sensitivity than reported herein could be achieved using our cost-optimized RT-qPCR protocol with fresh PBMCs, as cryopreservation can profoundly influence surface marker and antigen-specific T cell responses (5, 24). Additionally, we speculate that our cost-optimized assay can be readily adapted to a wide range of cell types. Moreover, although results were reported herein for only *IFN-γ, IL-2* and *TNF-α* mRNA, this assay can be readily adapted to a broad range of effector function markers by using different primer sets. When assessing cytokine expression kinetics, peak mRNA expression was stimulant, cytokine, and donor-dependent. To the best of our knowledge, ours is the first study of *IFN-γ*, *IL-2* and *TNF-α* mRNA expression kinetics post-stimulation with hourly resolution across a 12-hour timeframe. Despite this variability, when considering the correlation between *IFN-γ* mRNA gene expression and protein production, we defined 6 hours post *in vitro* stimulation as optimal for all donors and peptide epitopes.

RT-qPCR-based HTS protocols which screen a broad range of samples and targets have been previously described; including screens for anti-parasitic drugs (11), bioactive small molecules (14, 31), or disease diagnostics (32). Those assays are limited by the cost of generating the high-quality and high-purity sample required for optimal qPCR. We have previously demonstrated the automatable technologies used in this protocol produce high quality and quantity RNA and cDNA (9). Herein, we demonstrate no statistically significant loss in the correlation between mRNA and protein quantification post assay miniaturization and cost-optimization. We speculate that with automated technologies capable of accurate ultra-low volume (*i.e.*, <0.1uL) dispensing, both cost-optimization and miniaturization could be extended further.

When assessing the confusion matrix between RTqPCR and ELIspot results, we tested two thresholds of positivity: (i) all results above background for both assays (*i.e.*, mRNA >0 and ELIspot SFC/10^6^ >0), or (ii) a doubling of mRNA and more than 1 in 10,000 responding cells (*i.e.*, mRNA >1 and ELIspot SFC/10^6^ >100). The improved accuracy (*i.e.*, 73.3% vs 90.8%) observed when the threshold of positivity was increased suggests that more sophisticated strategies to define positivity (*i.e.*, statistical testing), or more stringent positivity criteria (*i.e.*, a change of 2 of more standard deviations), may further increase assay accuracy in larger screens. ELIspot is generally considered positive above a threshold (*i.e.*, 40-100 SFC/10^6^) (21). However, a threshold strategy for qPCR may overlook low level mRNA responses from antigen reactive cells. We expect that defining an experimental threshold of positivity for a RTqPCR-based HTS immune-assay will be dependent upon sample, stimulation, and desired experimental outcome.

We expect that this study will be of broad interest to a diverse number of researchers by facilitating comprehensive laboratory and field studies. One example where high-throughput functional immunoassays may provide critical experimental information is during vaccine candidate testing (33–35). This assay would allow more comprehensive preclinical or clinical studies, with either more samples or more parameters per sample, without requiring additional engineering or modification steps such as those required for luciferase or other luminescence-based reporter screens (36). Additionally, high-throughput transcriptome profiling of RNA-based biomarkers of disease have been reported for a broad range of malignancies including lung (37), skin (38) and breast (39) carcinomas, and other diseases such as rheumatoid arthritis (40). An assay with high analytical and diagnostic sensitivity which allows cost-efficient isolation and quantification of PBMC RNA is likely to be highly beneficial.

When considering transcription-based molecular diagnostics, RT-qPCR is highly sensitive and specific and is relatively cheap and uncomplicated to analyze (41, 42). Other transcription-based techniques include Northern blotting (43), *in-situ* hybridization (44), RNA microarrays (45), NanoString^™^ (46), Sanger and Next-Generation Sequencing (NGS) (47) and advanced PCR techniques (*e.g.*, digital PCR (48), microsphere-multiplex PCR (49)). Higher-throughput transcription-based techniques will likely surpass RT-qPCR in cost-efficiency. Still, SYBR^®^-chemistry will remain the most inexpensive option for effective sensitive and specific mRNA quantitation for the foreseeable future.

In conclusion, we present herein an HTS-compatible assay with high analytical and diagnostic sensitivity, which allows cost-efficient isolation and quantification of PBMC RNA. This robust, scalable, and cost-effective alternative to protein-based *ex vivo* PBMC immunoassays addresses the limitations of cost and sample volume associated with standard immunoassay protocols. By overcoming these well-accepted constraints, we anticipate this assay will have widespread applicability in preclinical and clinical studies (50), especially when samples are limited, and cost is an important consideration.

## Data availability statement

The original contributions presented in the study are included in the article/**Supplementary Material**. Further inquiries can be directed to the corresponding author.

## Ethics statement

The studies involving human participants were reviewed and approved by James Cook University Human Research Ethics Committee (#H7886). The patients/participants provided their written informed consent to participate in this study.

## Author contributions

DB and AK performed experiments. DB, JB, and DD designed experiments. DB, JB, AK, and DD analyzed and interpreted the data. DB and DD wrote the manuscript with input from JB and AK. All authors contributed to the article and approved the submitted version.

## Funding

Work was supported by the National Health and Medical Research Council of Australia (grant #1069466**)**. DD is supported by a NHMRC Principal Research Fellowship (#1137285). DB is supported by James Cook University Prestige Research Training Program Stipend (RTPS).

## Acknowledgments

We are grateful to the donors who kindly provided samples for study. We thank Drs. John Miles and Yide Wong for the kind gifts of ‘FLY’, ‘GLC’, and ‘CLG’ peptides. We thank Dr Corey Smith for providing us with the list of peptides. The graphical abstract was created with biorender.com.

## Conflict of interest

The authors declare that the research was conducted in the absence of any commercial or financial relationships that could be construed as a potential conflict of interest.

## Publisher’s note

All claims expressed in this article are solely those of the authors and do not necessarily represent those of their affiliated organizations, or those of the publisher, the editors and the reviewers. Any product that may be evaluated in this article, or claim that may be made by its manufacturer, is not guaranteed or endorsed by the publisher.
