# Supplementary material for: A high-throughput screening RT-qPCR assay for quantifying surrogate markers of immunity from PBMCs
Source: Front Immunol. 2022 Aug 30;13:962220. doi: 10.3389/fimmu.2022.962220 (PMC9469018; doi:10.3389/fimmu.2022.962220)
Supplement: Supplementary file 1 [file DataSheet_1.zip › Supplementary Document 1_Figures and Tables.docx]

Supplementary Material

**A high-throughput screening (HTS) RTqPCR assay for quantifying surrogate markers of immunity from PBMCs**

Daniel J. Browne, Ashton M. Kelly, Jamie L. Brady and Denise L. Doolan


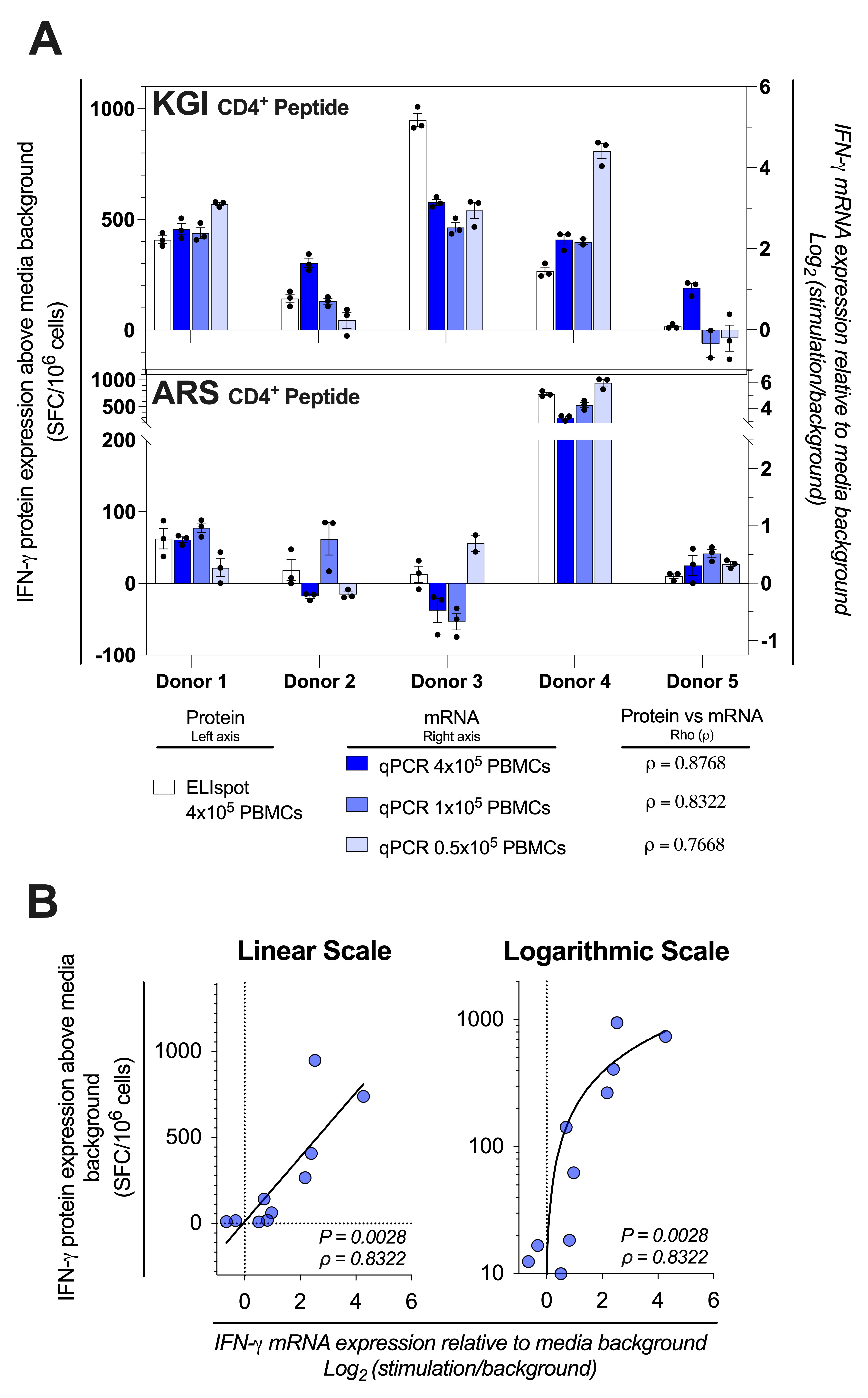


**Supplementary Figure 1. mRNA expression correlates with protein-based quantification following stimulation of PBMCs with CD4^+^ peptides. (a)** IFN-γ mRNA expression by RTqPCR compared to IFN-γ protein production by ELIspot following stimulation of PBMCs (4x10^5^, 1x10^5^ or 5x10^4^; n=5) with synthetic peptides representing two well-defined CD4^+^ T cell peptide-epitopes (Influenza_57-71_ KGILGFVFTLTVPSE and Influenza_260-284_ ARSALILRGSVAHKSCLPACVYGP). Spot forming cells (SFC) were quantified from technical triplicate stimulations by ELIspot. Gene copy number per reaction were quantified from single stimulations by absolute quantification RTqPCR. Single full volume RNA extractions, with single full volume reverse transcription reactions, and qPCR in technical triplicate replicates were performed as per manufacturers recommendation. The technical mean ± technical SEM of gene copy number or SFC corrected for background are shown. (**b**) IFN-γ mRNA expression by RTqPCR correlated to IFN-γ protein production by ELIspot graphed either on a linear or logarithmic scale, following stimulation of 1x10^5^ PBMCs (n=5). The strength of the association between RTqPCR IFN-γ mRNA gene expression (on Log_2_ transformed data) and ELIspot IFN-γ protein expression (linear data) was tested by Pearson’s correlation. *P* values and Pearson’s correlation coefficient (ρ) reported.

|  |
| --- |
| 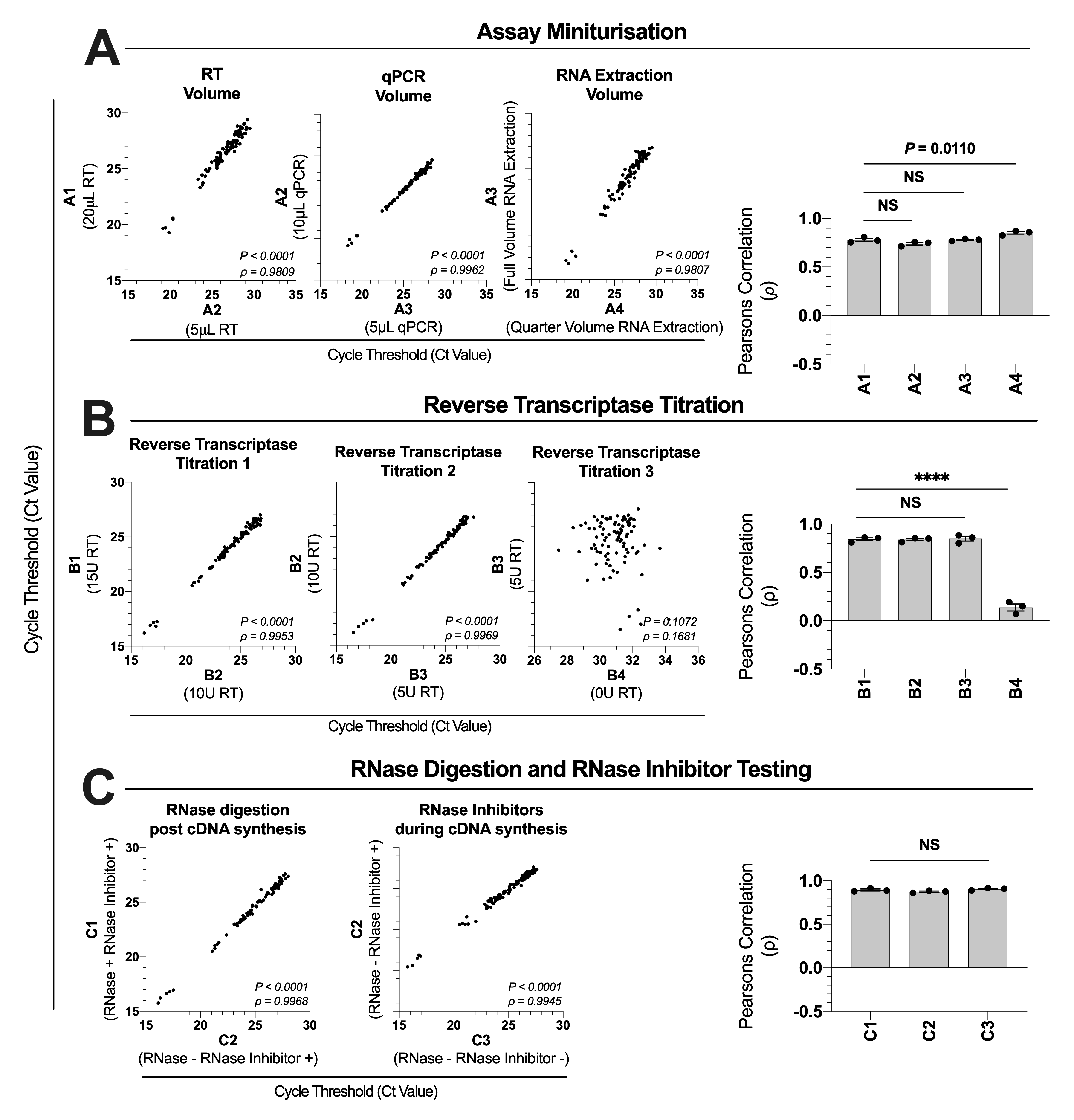 |
| **Supplementary Figure 2. Correlations between RTqPCR data following assay optimization.** IFN-γ mRNA expression by RTqPCR correlated to IFN-γ mRNA expression by RTqPCR following systematic adjustments the RTqPCR protocol (left panels); and the RT-qPCR assay was performed in technical triplicate, with each replicate individually correlated to the mean IFN-γ SFC by ELIspot (right panel) and tested using a One-Way ANOVA with a Bonferroni corrected multiple comparisons test. (**A**) A1, A2, A3 or A4 (top panel) were evaluating the correlation between 20 μl versus 5 μl final volume single reverse transcription (RT) reactions containing 20 units/μLRNA (20U) of reverse transcriptase enzyme; the correlation between 10 μl versus 5 μl final volume qPCR; and the correlation between full volume and quarter volume RNA extractions. All RT reactions included RNase Inhibitors and a post-cDNA synthesis RNase digestion stage. (**B**) B1, B2, B3 or B4 (middle panel) were evaluating the correlation between reverse transcription (RT) reactions containing 15 units/μL_RNA_ (15U) or 10 units/μL_RNA_ (10U) of reverse transcriptase enzyme; the correlation between RT reactions containing 10U versus 5U reverse transcriptase enzyme; and the correlation between RT reactions containing 5U versus 0U reverse transcriptase enzyme. All RT reactions included RNase Inhibitors and a post-cDNA synthesis RNase digestion stage. (**C**) C1, C2 and C3 (bottom panel) were evaluating the correlation between reverse transcription (RT) reactions including RNase Inhibitors and a post-cDNA synthesis RNase digestion stage, and reactions excluding the RNase digestion stage; and RT reactions excluding the RNase digestion stage and RT reactions excluding both the RNase digestion stage and RNase Inhibitors. The strength of the association between RTqPCR IFN-γ mRNA gene expression and ELIspot IFN-γ protein expression was tested by Pearson’s correlation. *P* values and Pearson’s correlation coefficient (ρ) reported. |
|  |
| 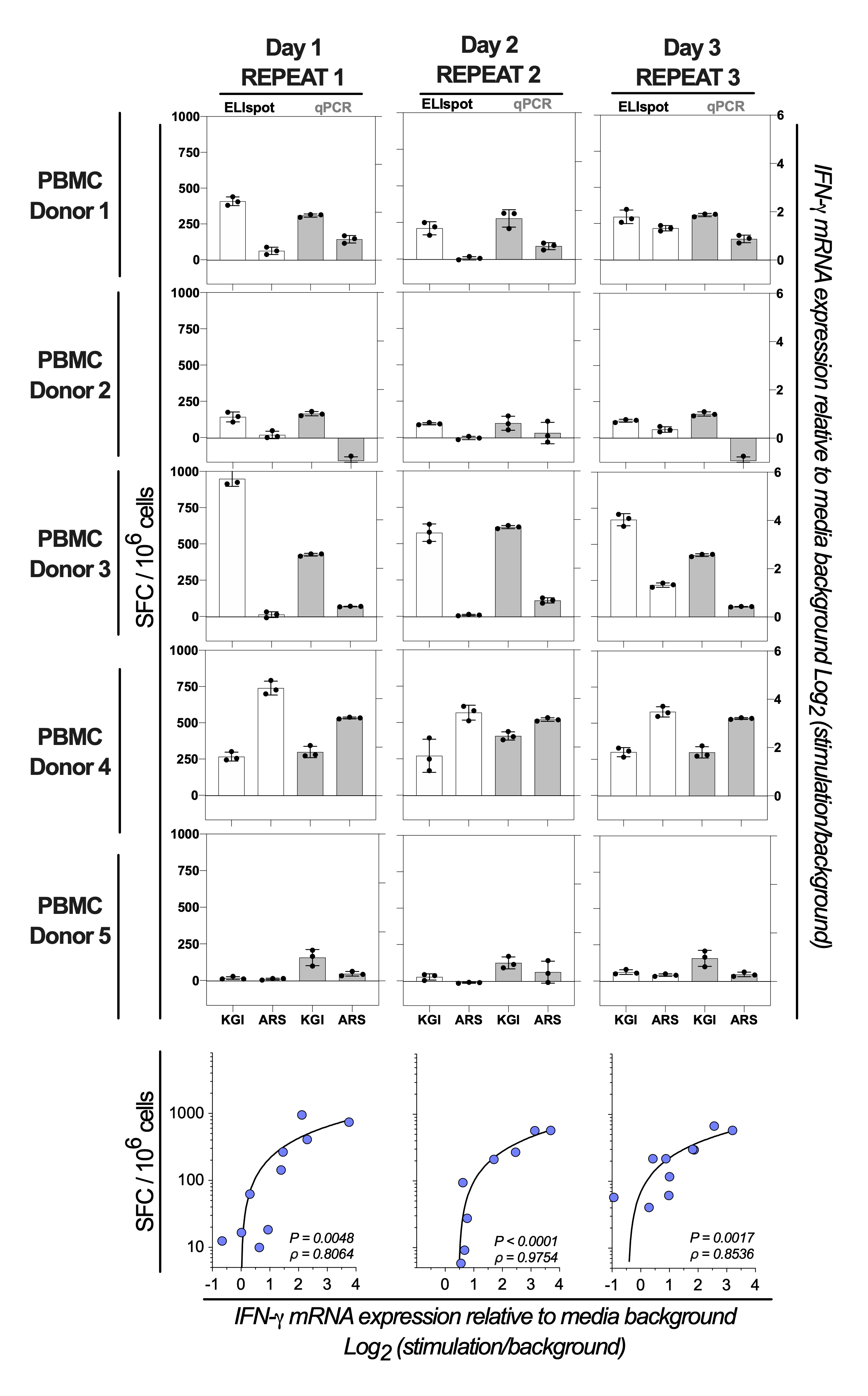 |
| **Supplementary Figure 3. Technical triplicate replicate inter-day testing.** IFN-γ mRNA expression (Grey Bars) by RTqPCR correlated to IFN-γ protein production (White Bars) by ELIspot following stimulation of PBMCs (1x10^5^; n=5) with peptides representing two well-defined CD4^+^ T cell peptide-epitopes (Influenza_57-71_ KGILGFVFTLTVPSE and Influenza_260-284_ ARSALILRGSVAHKSCLPACVYGP) across three inter-day technical replicate experiments. Spot forming cells (SFC) were quantified from technical triplicate stimulations by ELIspot. Gene copy number per reaction were quantified from single stimulations by absolute quantification RTqPCR. Quarter (manufacturers recommendation) volume RNA extractions, with single 5 ml final volume reverse transcription (RT) reactions containing 5 units/μL_RNA_ (5U) of reverse transcriptase enzyme per n, were performed. RT reactions were exclusive of RNase Inhibitors or a post-cDNA synthesis RNase digestion stage. 5 ml final volume qPCR was performed in technical triplicate replicate. Shown are the technical mean ± technical SEM of the technical triplicate gene copy number or spot forming cells (SFC) corrected for background. The strength of the association between RTqPCR IFN-γ mRNA gene expression and ELIspot IFN-γ protein expression was tested by Pearson’s correlation on log transformed data with *P* values and Pearson’s correlation coefficient (ρ) reported. SFC≤20/1x10^6^ PBMCs were omitted when correlating protein to mRNA. The correlation of each technical replicate was tested with One-Way ANOVA with a Bonferroni corrected multiple comparisons test reported |

**

**

**Supplementary Figure 4. Cytokine mRNA expression kinetics in PBMCs in response to antigen and mitogen stimulation.** Hourly kinetics of *IFN-γ*, *TNF-a* and *IL-2* mRNA expression from 1x10^5^ PBMCs from three individuals (PBMC Donor 1, 2 and 3) stimulated with MHC Class I CD8^+^ (GIL; pink cross, GLC; pink square and FLY pink triangle) or MHC Class II CD4^+^ (KGI; blue cross, and ARS; blue square) peptides. Mean of gene copy number relative to background are shown. Single RNA extractions, with single reverse transcription reactions per n per stimulation were performed, with qPCR performed in technical triplicate replicates. Gene copy number per reaction was quantified by absolute quantification. The sample mean was calculated from the mean of the technical triplicate replicates. All samples (n=3) were stimulated with individual peptide in parallel with media negative background control and CEF peptide pool and PMA/Iono positive controls.

**
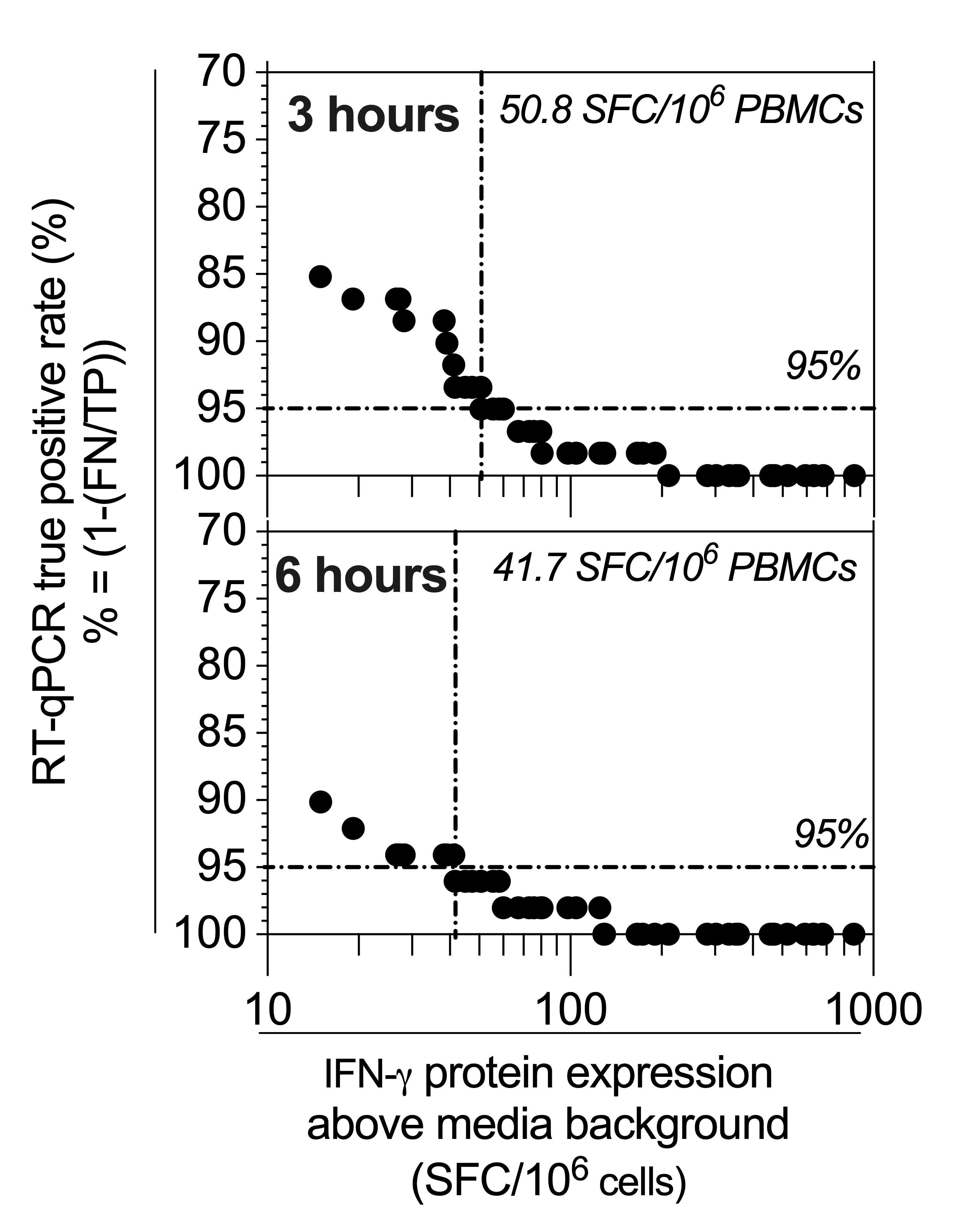
**

**Supplementary Figure 5. The true-positive rate of RTqPCR compared to ELIspot at 3- and 6-hours.** The true positive rate of the RT-qPCR assay was calculated as the inverse of RT-qPCR total false negatives (FN) relative to true positives (TP) compared to ELIspot. 95% diagnostic sensitivity threshold and time specific diagnostic sensitivity shown - dashed line.

**
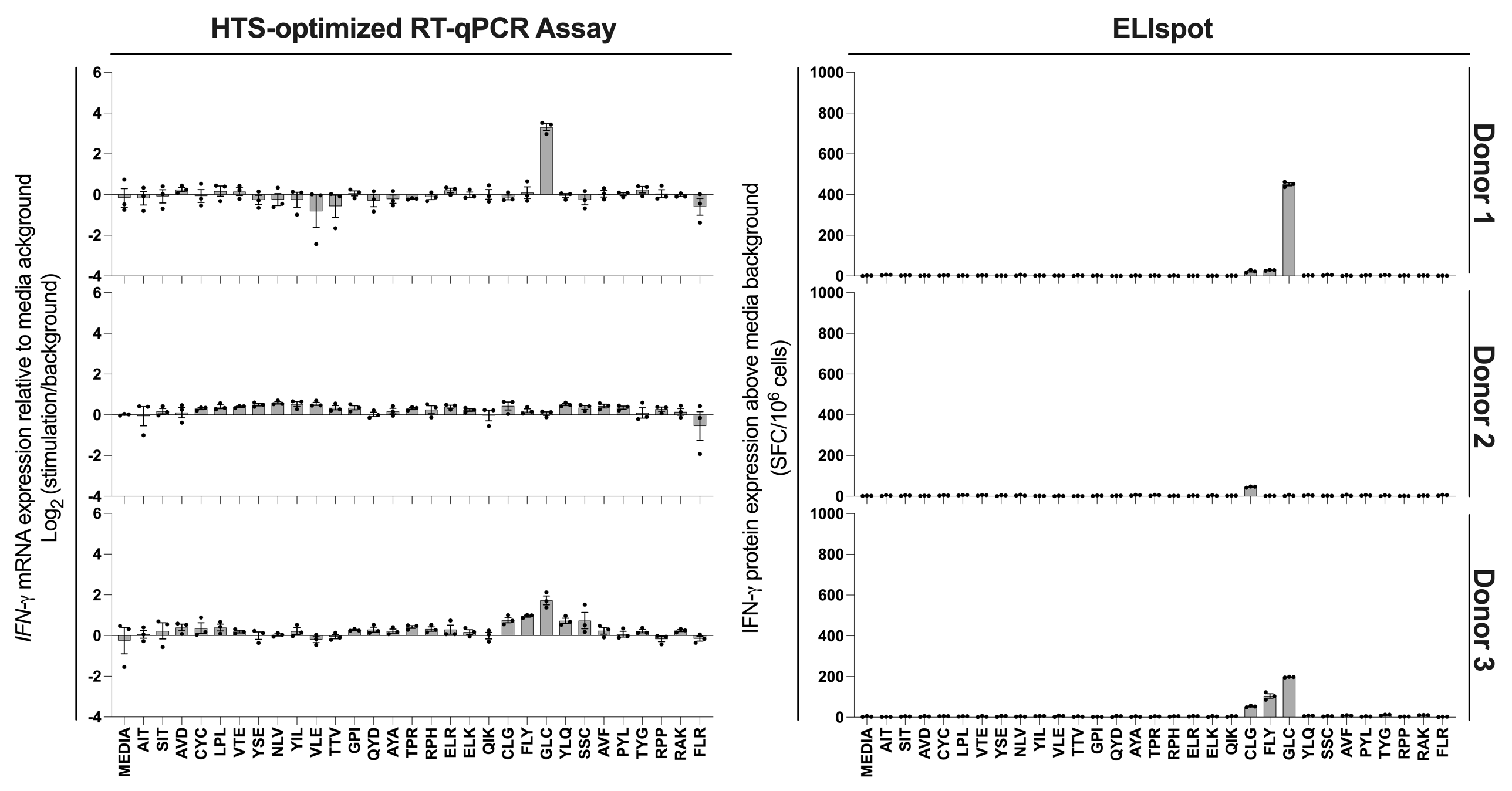
**

**Supplementary Figure 6. The response to stimulation measuring a broad number of peptides epitopes.** IFN-γ mRNA expression by high-throughput screening (HTS) optimized RT-qPCR and IFN-γ protein production by ELIspot following stimulation with 30x well-defined CD8^+^ peptide epitope stimulations of PBMCs (n=3). Shown are the mean ± technical SEM of gene copy number of technical triplicate RT-qPCR assays, and the mean ± technical SEM of triplicate IFN-γ spot forming cells (SFC) by ELIspot, with both mRNA and protein measurements corrected for background.

| **Supplementary Table 1. Synthetic peptides.** Peptides representing defined CD8^+^ or CD4^+^ T cell peptide epitopes derived from Epstein Barr Virus and Influenza Virus; and Anaspec CEF control peptide pool (CEFpp) representing 32 CD8^+^ T cell peptide epitopes derived from human Cytomegalovirus, Epstein-Barr Virus or Influenza Virus. | | | | | | |
| --- | --- | --- | --- | --- | --- | --- |
| **Code** | **Epitope** | **Species Derived** | **Antigen** | **Residues** | **MHC Class / T cell** | **Reference** |
| CEFpp | Multiple^*^ | Cytomegalovirus  Epstein-Barr Virus  Influenza Virus | Multiple* | Multiple* | MHC I / CD8^+^ | [1] |
| KGI | KGILGFVFTLTVPSE | Influenza virus | Matrix protein 1 | 57-71 | MHC II / CD4^+^ | [2] |
| ARS | ARSALILRGSVAHKSCLPACVYGP | Influenza virus | Nucleoprotein | 260-284 | MHC II / CD4^+^ | [3] |
| GIL | GILGFVFTL | Influenza virus | Matrix protein 1 | 58-76 | MHC I / CD8^+^ | [4, 5] |
| AIT | AITEVECFL | Polymaviruses | VP1 | 44-53 | MHC I / CD8^+^ | [6] |
| SIT | SITEVECFL | Polymaviruses | VP1 | 36-44 | MHC I / CD8^+^ | [6] |
| AVD | AVDTVLAKK | Polymaviruses | Large T antigen | 341-349 | MHC I / CD8^+^ | [7] |
| CYC | CYCIDCFTQW | Polymaviruses | Small T antigen | 136-145 | MHC I / CD8^+^ | [7] |
| LPL | LPLMRKAYL | Polymaviruses | Large T antigen | 27-35 | MHC I / CD8^+^ | [7] |
| VTE | VTEHDTLLY | Cytomegalovirus | pp50 | 245-253 | MHC I / CD8^+^ | [8] |
| YSE | YSEHPTFTSQY | Cytomegalovirus | pp65 | 363–373 | MHC I / CD8^+^ | [9] |
| NLV | NLVPMVATV | Cytomegalovirus | pp65 | 495–503 | MHC I / CD8^+^ | [9] |
| YIL | YILEETSVML | Cytomegalovirus | IE-1 | 315-324 | MHC I / CD8^+^ | [10] |
| VLE | VLEETSVML | Cytomegalovirus | IE-1 | 316–324 | MHC I / CD8^+^ | [9] |
| TTV | TTVYPPSSTAK | Cytomegalovirus | pp150 | 947-957 | MHC I / CD8^+^ | [11] |
| GPI | GPISGHVLK | Cytomegalovirus | pp65 | 16-25 | MHC I / CD8^+^ | [11] |
| QYD | QYDPVAALF | Cytomegalovirus | pp65 | 328-337 | MHC I / CD8^+^ | [12] |
| AYA | AYAQKIFKIL | Cytomegalovirus | IE-1 | 248-258 | MHC I / CD8^+^ | [12] |
| TPR | TPRVTGGGAM | Cytomegalovirus | pp65 | 417–426 | MHC I / CD8^+^ | [9] |
| RPH | RPHERNGFTVL | Cytomegalovirus | pp65 | 265-275 | MHC I / CD8^+^ | [13] |
| ELR | ELRRKMMYM | Cytomegalovirus | IE-1 | 199–207 | MHC I / CD8^+^ | [14] |
| ELK | ELKRKMIYM | Cytomegalovirus | IE-1 | 199-207 | MHC I / CD8^+^ | [9] |
| QIK | QIKVRVDMV | Cytomegalovirus | IE-1 | 88–96 | MHC I / CD8^+^ | [9] |
| FLY | FLYALALLL | Epstein Barr Virus | LMP2 | 356-364 | MHC I / CD8^+^ | [15] |
| GLC | GLCTLVAML | Epstein Barr Virus | BMLF1 | 280–288 | MHC I / CD8^+^ | [16] |
| CLG | CLGGLLTMV | Epstein Barr Virus | LMP2 | 426-434 | MHC I / CD8^+^ | [17] |
| YLQ | YLQQNWWTL | Epstein Barr Virus | LMP1 | 159-167 | MHC I / CD8^+^ | [18] |
| SSC | SSCSSCPLSKI | Epstein Barr Virus | LMP2 | 340–350 | MHC I / CD8^+^ | [19] |
| AVF | AVFDRKSDAK | Epstein Barr Virus | BERF2a | 399-428 | MHC I / CD8^+^ | [20] |
| PYL | PYLFWLAAI | Epstein Barr Virus | LMP2 | 131-139 | MHC I / CD8^+^ | [19] |
| TYG | TYGPVFMCL | Epstein Barr Virus | LMP2 | 419-427 | MHC I / CD8^+^ | [17] |
| RPP | RPPIFIRRL | Epstein Barr Virus | EBNA 3A | 881-889 | MHC I / CD8^+^ | [21] |
| RAK | RAKFKQLL | Epstein Barr Virus | BZLF1 | 190–197 | MHC I / CD8^+^ | [22] |
| FLR | FLRGRAYGL | Epstein Barr Virus | EBNA3A | 325–333 | MHC I / CD8^+^ | [22] |
| LMP2: Latent Membrane Protein 2; BMLF1: mRNA export factor ICP27 homolog; pp50: 50K phosphoprotein; VP1: Major capsid protein VP1; pp65: 65K phosphoprotein; IE-1: Immediate Early Prottein 1; 150K phosphoprotein; LMP1: Latent Membrane Protein 1; BERF2a: BamHI-E rightward reading frames 2a; EBNA-3A: nuclear antigen EBNA-3A; BZLF1: BamHI Z fragment leftward open reading frame 1  *32 x 8-12 amino acids in length | | | | | | |
|  | | | | | | |

|  | | | | | |  |  |
| --- | --- | --- | --- | --- | --- | --- | --- |
| **Supplementary Table 2. Oligonucleotide primer list.** Characteristics of primers acquired from the PrimerBank^™^ database. Primers were evaluated with logarithmically diluted PBMC cDNA wherein reaction efficiency (E′) and standard coefficient of determination (R^2^) were determined per MIQE guidelines. | | | | | | | |
| **Transcript** | **GenBank Accession Number** | **PrimerBank**^™^  **ID^*^** | **Forward Sequence**  (5’-3’) | **Reverse Sequence**  (5’-3’) | **Amplicon Size**  (bp) | **E’**  (%) | **R^2^** |
| *RPL13a* | NM_012423 | 14591905c2 | GCCCTACGACAAGAAAAAGCG | TACTTCCAGCCAACCTCGTGA | 117 | 94.5 | 0.99 |
| *IFN-γ* | NM_000619.2 | 56786137c1 | TCGGTAACTGACTTGAATGTCCA | TCGCTTCCCTGTTTTAGCTGC | 93 | 99.7 | 0.99 |
| *TNFα* | NM_000594 | 25952110c2 | GAGGCCAAGCCCTGGTATG | CGGGCCGATTGATCTCAGC | 91 | 99.3 | 0.99 |
| *IL-2* | NM_000586.3 | 28178861a1 | AACTCCTGTCTTGCATTGCAC | GCTCCAGTTGTAGCTGTGTTT | 93 | 91.2 | 0.99 |
| * https://pga.mgh.harvard.edu/primerbank/ | | | | | |  |  |

**References**

1. Sedegah, M., et al., *Sterile immunity to malaria after DNA prime/adenovirus boost immunization is associated with effector memory CD8+T cells targeting AMA1 class I epitopes.* PLoS One, 2014. **9**(9): p. e106241.

2. Sheikh, Q.M., et al., *Towards the knowledge-based design of universal influenza epitope ensemble vaccines.* Bioinformatics, 2016. **32**(21): p. 3233-3239.

3. Gao, X.M., F.Y. Liew, and J.P. Tite, *Identification and characterization of T helper epitopes in the nucleoprotein of influenza A virus.* J Immunol, 1989. **143**(9): p. 3007-14.

4. Choo, J.A., et al., *The immunodominant influenza A virus M158-66 cytotoxic T lymphocyte epitope exhibits degenerate class I major histocompatibility complex restriction in humans.* J Virol, 2014. **88**(18): p. 10613-23.

5. Gotch, F., et al., *Cytotoxic T lymphocytes recognize a fragment of influenza virus matrix protein in association with HLA-A2.* Nature, 1987. **326**(6116): p. 881-2.

6. Chen, Y., et al., *Interplay of cellular and humoral immune responses against BK virus in kidney transplant recipients with polyomavirus nephropathy.* J Virol, 2006. **80**(7): p. 3495-505.

7. Ambalathingal, G.R., et al., *Proteome-wide analysis of T-cell response to BK polyomavirus in healthy virus carriers and kidney transplant recipients reveals a unique transcriptional and functional profile.* Clin Transl Immunology, 2020. **9**(1): p. e01102.

8. Giest, S., et al., *Cytomegalovirus-specific CD8+ T cells targeting different peptide/HLA combinations demonstrate varying T-cell receptor diversity.* Immunology, 2012. **135**(1): p. 27-39.

9. Khan, N., et al., *Persistent viral infection in humans can drive high frequency low-affinity T-cell expansions.* Immunology, 2010. **131**(4): p. 537-48.

10. Prod'homme, V., et al., *Modulation of HLA-A*0201-restricted T cell responses by natural polymorphism in the IE1(315-324) epitope of human cytomegalovirus.* J Immunol, 2003. **170**(4): p. 2030-6.

11. Quinzo, M.J., et al., *Computational assembly of a human Cytomegalovirus vaccine upon experimental epitope legacy.* BMC Bioinformatics, 2019. **20**(Suppl 6): p. 476.

12. Kuzushima, K., et al., *Efficient identification of HLA-A*2402-restricted cytomegalovirus-specific CD8(+) T-cell epitopes by a computer algorithm and an enzyme-linked immunospot assay.* Blood, 2001. **98**(6): p. 1872-81.

13. Huth, A., et al., *Antigen-Specific TCR Signatures of Cytomegalovirus Infection.* J Immunol, 2019. **202**(3): p. 979-990.

14. Tey, S.K., F. Goodrum, and R. Khanna, *CD8+ T-cell recognition of human cytomegalovirus latency-associated determinant pUL138.* J Gen Virol, 2010. **91**(Pt 8): p. 2040-2048.

15. Lautscham, G., et al., *Identification of a TAP-independent, immunoproteasome-dependent CD8+ T-cell epitope in Epstein-Barr virus latent membrane protein 2.* J Virol, 2003. **77**(4): p. 2757-61.

16. Bharadwaj, M., et al., *Contrasting Epstein-Barr virus-specific cytotoxic T cell responses to HLA A2-restricted epitopes in humans and HLA transgenic mice: implications for vaccine design.* Vaccine, 2001. **19**(27): p. 3769-77.

17. Lin, X., et al., *Chimerically fused antigen rich of overlapped epitopes from latent membrane protein 2 (LMP2) of Epstein-Barr virus as a potential vaccine and diagnostic agent.* Cell Mol Immunol, 2016. **13**(4): p. 492-501.

18. Khanna, R., et al., *Identification of cytotoxic T cell epitopes within Epstein-Barr virus (EBV) oncogene latent membrane protein 1 (LMP1): evidence for HLA A2 supertype-restricted immune recognition of EBV-infected cells by LMP1-specific cytotoxic T lymphocytes.* Eur J Immunol, 1998. **28**(2): p. 451-8.

19. Straathof, K.C., et al., *Characterization of latent membrane protein 2 specificity in CTL lines from patients with EBV-positive nasopharyngeal carcinoma and lymphoma.* J Immunol, 2005. **175**(6): p. 4137-47.

20. Khanna, R., et al., *Molecular characterization of antigen-processing function in nasopharyngeal carcinoma (NPC): evidence for efficient presentation of Epstein-Barr virus cytotoxic T-cell epitopes by NPC cells.* Cancer Res, 1998. **58**(2): p. 310-4.

21. Hill, A., et al., *Characterization of two Epstein-Barr virus epitopes restricted by HLA-B7.* Eur J Immunol, 1995. **25**(1): p. 18-24.

22. Falco, D.A., et al., *Identification of Epstein-Barr virus-specific CD8+ T lymphocytes in the circulation of pediatric transplant recipients.* Transplantation, 2002. **74**(4): p. 501-10.
