# Supplementary material for: A high-throughput screening RT-qPCR assay for quantifying surrogate markers of immunity from PBMCs
Source: Front Immunol. 2022 Aug 30;13:962220. doi: 10.3389/fimmu.2022.962220 (PMC9469018; doi:10.3389/fimmu.2022.962220)
Supplement: Supplementary file 1 [file DataSheet_1.zip › Supplementary Document 2_Supp Protocol.docx]

**Supplementary Protocol: Cost-Optimized Protocol:**

The below protocol complies with ‘Protocol Exchange’ formatting

<https://protocolexchange.researchsquare.com/>

**Introduction:**

Herein we present a reverse-transcription quantitative PCR (RTqPCR) assay specifically designed to provide a high-throughput, robust, scalable, and cost-effective alternative to protein-based *in vitro* immunoassays. This protocol is targeted towards high-throughput studies of antigen-specific immune responses in human peripheral blood mononuclear cells (PBMCs). This assay is amendable to miniaturization and automation, exceeds HTS uniformity and signal variance testing standards, and represents a cost saving of almost 90% on standard-practice assays. This HTS-optimized protocol has single-cell analytical sensitivity and a diagnostic sensitivity equivalent to detecting 1:10,000 responding cells (*i.e.,* 100 Spot Forming Cells/10^6^ PBMCs by ELIspot) with 90% accuracy.

**Reagents:**

1. Molecular grade isopropanol (Sigma-Aldrich: Catalog# I9516)
2. Molecular grade ethanol (Sigma-Aldrich: Catalog# E7023)
3. Molecular grade β-Mercaptoethanol (Sigma-Aldrich: Catalog# M3148)
4. MagMAX^™^ *mirVana*^™^ Total RNA isolation Kit (ThermoFisher: Catalog# A27828)
5. Superscript IV^™^ Enzyme kit (ThermoFisher: Catalog# 18090050)
6. 50 µM Random Hexamers (ThermoFisher: Catalog# N8080127)
7. 10 mM dNTPs (ThermoFisher: Catalog# R0191)
8. Ultra-Pure H_2_O (ThermoFisher: Catalog# 10977015)
9. ssoAdvanced SYBR^™^ (BioRad: Catalog# 1725270)
10. RNaseZAP^™^ (Sigma-Aldrich: Catalog# R2020)
11. Desalt Grade qPCR Primers (Sigma-Aldrich) (<https://pga.mgh.harvard.edu/primerbank/>)

**Equipment:**

1. 96-well U-bottom incubation plate (Falcon: Catalog# 353077)
2. CO_2_ incubator: (LabGear: SCO6AD-2)
3. Benchtop Centrifuge (Beckman Coulter: Avanti J-15R)
4. p200 multichannel pipette (Eppendorf: Research Plus)
5. p10, p20, p200 and p1000 pipettes (Eppendorf: Research Plus)
6. Microplate shaker (GrantBio: PMS 100i)
7. Magnetic stand (Invitrogen: Catalog# AM10027)
8. Vortex mixer (GrantBio: PV1)
9. Benchtop mini-centrifuge (LabGear: LABG3000001)
10. 0.2 mL PCR-grade clean tube (Eppendorf: PCR grade)
11. Thermocycler (SimpliAmp™ Thermal Cycler: Catalog# A24811)
12. MicroAmp^™^ Optical 384-Well Reaction Plate (Thermo Fisher: Catalog# 4309849)
13. MicroAmp^™^ Optical Adhesive Film (Thermo Fisher: Catalog# 4311971)
14. QuantStudio^™^ 5 Quantitative Thermocycler (Applied Biosystems)
15. QuantStudio^™^ 5 Design and Analysis Software (Applied Biosystems)

**Procedure:**

1. **Experimental cell stimulation/culture**
   1. Stimulate or culture PBMCs as per standard practice in a 96-well U-bottom incubation plate/s
      1. This assay is suitable for testing PBMCs between 1x10^6^ – 1x10^0^ PBMCs per well.
      2. A higher diagnostic sensitivity would be expected with a higher PBMC cell input.
      3. For interferon-gamma (IFN-γ) expression, 1x10^5^ PBMCs have a diagnostic sensitivity equivalent to 100/10^6^ spot forming cells (SFC/10^6^) by ELIspot, with a accuracy of 90%
      4. Peak mRNA expression is stimulant and individual dependent, however 6-hours post-stimulation is generally optimal for correlating mRNA to protein
   2. Prepare ‘*Lysis Binding Mix*’ as per Protocol Table 1.

| **Protocol Table 1. Lysis Binding Mix** | | |
| --- | --- | --- |
| **Component** | **Amount**  **(1x)** | **Amount**  **(____x)** |
| Lysis Buffer | 25 μL |  |
| Molecular Grade Isopropanol | 25 μL |  |
| β-Mercaptoethanol | 1 μL |  |
| Total | 51 μL |  |

- 1. Centrifuge the 96-well U-bottom incubation plate/s at 1000 x g for 4 min
  2. Remove the media, and lyse cells in 50 μL of the *Lysis Binding Mix* directly in the U-bottom incubation plate
     1. Do not disturb the cell pellet until lysis step.
     2. Remove as much media as possible
     3. Mechanical lysis can be achieved by pipetting up and down 5x

(The cell pellet will almost immediately dissolve).

- - 1. **STOP POINT** – Cell lysate can be stored in -80°C for 2 weeks without RNA degradation. When thawing from -80^o^C, thaw samples at 4^o^C (on ice).

1. **RNA Extraction**
   - 1. To decontaminate the working area, gently wipe down all surfaces and equipment with lint free, non-woven, non-abrasive wipes sprayed with RNaseZAP^™^
     2. Preheat Elution Buffer to 37^o^C
     3. Prepare ‘*TURBO DNase solution*’ and ‘*Bead Binding Mix*’ as per Protocol Table 2 and 3 respectively.

| **Protocol Table 2. TURBO DNase solution** | | |
| --- | --- | --- |
| **Component** | **Amount**  **(1x)** | **Amount**  **(____x)** |
| MagMAX TURBO DNase Buffer | 12 μL |  |
| TURBO DNase | 0.5 μL |  |
| Total | 12.5 μL |  |

| **Protocol Table 3. Bead Binding Mix** | | |
| --- | --- | --- |
| **Component** | **Amount**  **(1x)** | **Amount**  **(____x)** |
| RNA Binding Beads | 2.5 μL |  |
| Lysis/Binding Enhancer | 2.5 μL |  |
| Total | 5 μL |  |

- 1. Cover and shake the U-bottom incubation plate on the microplate shaker for 3 min at speed 7 (700 rpm), to ensure samples are homogenous and are at room temperature.
  2. Add 5 μL ‘Binding Bead Mix’ to each sample
  3. Cover and shake the U-bottom incubation plate on the microplate shaker for 5 min at speed 6 (600 rpm)
  4. Place the U-bottom incubation plate on the magnetic stand for 5 min
     1. The solution will clear
  5. Carefully aspirate and discard the supernatant
     1. Do not to disturb the RNA binding beads
  6. Remove the U-bottom incubation plate from the magnetic stand
  7. Add 50 μL of MagMAX^™^ Wash Solution 1 to each sample
  8. Cover and shake the U-bottom incubation plate on the microplate shaker for 1 min at speed 7 (700 rpm)
  9. Place the U-bottom incubation plate on the magnetic stand for 1 min
     1. The solution will clear
  10. Repeat steps f. to j. to perform a second 50 μL wash with MagMAX^™^ Wash Solution 2
  11. Carefully aspirate and discard supernatant without disturbing the RNA binding beads
  12. Shake (the uncovered) U-bottom incubation plate for 2 min at speed 12 (1200 rpm), to evaporate residual ethanol
      1. Be careful not to over dry the beads
  13. Add 12.5 μL of ‘TURBO DNase Solution’ to each sample
  14. Cover and shake the U-bottom incubation plate for 15 min at speed 11 (1100 rpm)
  15. Add 12.5 μL MagMAX^™^ Rebinding Buffer
  16. Add 25 μL molecular grade isopropanol
      1. NEVER premix these reagents – pre mixing these reagents will cause the RNA extraction to fail
  17. Cover and shake U-bottom incubation plate for 3 min at speed 7 (700 rpm)
  18. Place the U-bottom incubation plate on the magnetic stand for 3 min
      1. The solution will clear
  19. Carefully aspirate and discard the supernatant
      1. Ensure not to disturb the RNA binding beads
  20. Remove the U-bottom incubation plate from the magnetic stand
  21. Add 50 μL of MagMAX^™^ Wash Solution 2 to each sample
  22. Cover and shake U-bottom incubation plate for 3 min at speed 7 (700 rpm)
  23. Place the U-bottom incubation plate on the magnetic stand for 1 min
      1. The solution will clear
  24. Repeat steps t. to x. to perform a second 50 μL wash with MagMAX^™^ Wash Solution 2
  25. Carefully aspirate and discard the supernatant
      1. Ensure not to disturb the RNA binding beads
  26. Shake (the uncovered) U-bottom incubation plate for 2 min at speed 10 (1200 rpm)
      1. This is to dry the plate by evaporation
      2. Be careful not to over dry the beads
  27. Add 15 μL of (preheated) MagMAX^™^ Elution Buffer to each sample
      1. 10 μL can be added to increase RNA concentration
  28. Cover and shake U-bottom incubation plate for 3 min at speed 11 (1100 rpm)
  29. Place the plate on the magnetic stand for 3 min
      1. The solution will clear
      2. The clear supernatant contains the samples RNA
      3. **STOP POINT** – Transfer supernatant to a fresh 96 well RNase Free 96 well plate for storage at -80^o^C. When thawing from -80^o^C, thaw samples at 4^o^C (on ice).

1. **cDNA Synthesis**
   - 1. Accurate and precise pipetting is critical
     2. Before beginning, gently wipe down all surfaces and equipment with lint free, non-woven, non-abrasive wipes sprayed with RNaseZAP
   1. Thaw and prepare the 50 µM Random Hexamers, 10 mM dNTPs, and the reagents within the Superscript IV™ Enzyme kit
      1. Thaw reagents on ice to minimize degradation
      2. Once defrosted, briefly vortex and centrifuge all defrosted reagents to ensure well mixed
2. Make up ‘*Primer Binding Mix (MM#1)*’ as per Protocol Table 4.

| **Protocol Table 4. Primer Binding Mix (MM#1)** | | |
| --- | --- | --- |
| **Component** | **1x** | **__x** |
| 50 µM Random Hexamers | 0.25 μL |  |
| H_2_O | 0.25 μL |  |
| 10 mM dNTP | 0.25 μL |  |
| Total | 0.75 μL |  |

1. Add 0.75 μL of ‘Primer Binding Mix (MM#1)’ per 0.2 mL PCR Tube
2. Add 2.5 μL of eluted RNA to each tube
   - 1. Normalization to RNA concentration is not required.
3. Nano-spectroscopy readings are not required and are likely to highly inaccurate as RNA concentrations will be very low (<10 ng/μL)
4. Incubate tube at 65^o^C for 5 minutes then cool to 4^o^C for at least 1 minute
5. Prepare ‘*cDNA Synth Mix (MM#2)*’ as per Protocol Table 5

| **Protocol Table 5. cDNA Synth Mix (MM#2)** | | |
| --- | --- | --- |
|  | **5U** | |
| **Component** | **1x** | **__x** |
| 5 x Superscript IV^™^ Buffer | 1 μL |  |
| 100 mM DTT | 0.25 μL |  |
| Superscript IV^™^ Enzyme | 0.063 μL |  |
| DEPC H_2_O | 0.438 μL |  |
| Total | 1.75 μL |  |

- - 1. A negative reverse transcription control mastermix can be made by substituting the Superscript IV^™^ enzyme with DEPC H_2_O

1. Add 1.75 μL of ‘*cDNA Synth Mix (MM#2)*’ to each tube
2. Incubate in ThermoCycler as per Protocol Table 6.

| **Protocol Table 6. cDNA synthesis conditions** | |
| --- | --- |
| Process | Conditions |
| Primer Priming and RT | 10 min at 23^o^C |
|  | 10 min at 50^o^C |
| Reaction Termination | 10 min at 85^o^C |

1. cDNA is now ready for use
   1. **STOP POINT** – cDNA can be stored long-term at -20^o^C
   2. Final cDNA volume = 5 μL
2. **Quantitative PCR**
   - 1. Accurate and precise pipetting is critical
   1. Dilute the cDNA 1:4 with Ultra-Pure H_2_O
      1. Final volume = 20 μL
   2. If performing absolute quantification prepare standard curve as per standard practice
      1. Standard curves can be made from either a plasmid containing the gene of interest, or purified amplicon.
   3. Mix primers into a forward and reverse working solution where each primer is at a 5 μM concentration (F&R 5 μM Primers)
   4. Prepare ‘*qPCR mastermix*’ as per standard qPCR practice using the below table.
      1. Ensure enough mastermix is made for the standards, a positive control, a no-template control and triplicate replicates for each cDNA sample.

| **Protocol Table 7. qPCR Mastermix** | | |
| --- | --- | --- |
| Mastermix | 1x | ___ x |
| 2 x ssoAdvanced SYBR supermix^™^ | 2.5 μL |  |
| 5 μM F&R Primers | 0.5 μL |  |
| Ultra-Pure H2O | 1 μL |  |
| Total | 4 μL |  |

- 1. Aliquot 4 μL of mastermix into each reaction well MicroAmp^™^ optical 384-well reaction plate
  2. Add 1 μL of sample (cDNA, standards, or controls) to each well
  3. Cover and seal the MicroAmp^™^ optical 384-well reaction plate with MicroAmp^™^ optical adhesive film
  4. Centrifuge 384-well reaction plate at 200 g for 1 second in the benchtop centrifuge
  5. Run and collect data as per ssoAdvanced SYBR supermix^™^ protocol and quantitative thermocycler as per standard practice
     1. If performing absolute quantification, quantitate copies per reaction.
     2. If performing relative quantification, quantitate copies per copy of reference gene.

1. **Data Analysis**
   1. Data is presented as ‘mRNA expression relative to media background’
   2. From the triplicate replicates of the negative control calculate the average (AVG_NEG_) individually for each sample
      1. For each technical qPCR replicate (x) calculate:

Log_2_ (x / AVG_NEG_)

- - 1. A value of 0 is equal to the mean of the negative media-only control

**Troubleshooting:**

**RNA extraction -** Poor RNA recovery is most commonly associated with allowing the magnetic beads to over dry; to avoid this issue, ensure a quick workflow. Another common problem is adding too much sample (*i.e.,* too many cells) into the wells. This assay has been tested for between 10^0^-10^6^ PBMCs per well of the U-bottom plate. Adding other tissue types or increased number of cells should be carefully optimized.

**cDNA Synthesis -** We report herein that reducing the concentration of the Superscript IV^™^ enzyme by 75% to 5 U/μL_RNA_ retains high analytical and diagnostic sensitivity when generating cDNA from 10^5^ PBMCs. It is likely, however, that higher enzyme concentrations will be required when synthesizing cDNA from samples containing inhibitors or a more concentrated RNA template.

**SYBR chemistry qPCR -** The most significant problem facing qPCR data is reproducibility across experiments and laboratories. To minimize this issue, all qPCR should be conducted and reported as per MIQE Guidelines. The protocol described herein allows normalization of data to PBMC number with absolute quantification. We promote absolute quantification when testing distinct cell numbers (e.g., PBMCs/condition). This strategy does not require reference genes for normalization and therefore eliminates issues associated with inappropriate reference gene selection. However, we stress that all MIQE guidelines must be followed to reduce the risk of making a type I or type II statistical error. This protocol would also be suitable when relative quantification is required (*e.g.,* incubations requiring cell proliferation).

**Time Taken:**

PBMC Stimulation/culture: 6 hours

RNA extractions: 96 samples can be extracted in 2 hours

cDNA synthesis: 96 samples can be prepped in 2 hours

SYBR qPCR: A 384 well plate can be loaded in 2 hours (and will take 1 hour to run with ssoAdvanced SOP on the quantitative thermocycler)

**Anticipated results:**

If the experiment is performed correctly as described above, users should be able to quantify total copies of mRNA target and compare increased or decreased expression relative to media controls.
